# Exploiting the Applicability of Polytetrahydrofuran-Modified Polyester for the Fabric Phase Sorptive Extraction of Doxycycline from Human Urine

**DOI:** 10.3390/molecules29174076

**Published:** 2024-08-28

**Authors:** Panagiotis Chatzintounas, Marianna Ntorkou, Abuzar Kabir, Constantinos K. Zacharis

**Affiliations:** 1 Laboratory of Pharmaceutical Analysis, Department of Pharmacy, Aristotle University of Thessaloniki, GR-54124 Thessaloniki, Greece; pchatzint@pharm.auth.gr (P.C.); marianna.ntorkou98@gmail.com (M.N.); 2 Department of Chemistry and Biochemistry, Florida International University, Miami, FL 33199, USA; akabir@fiu.edu

**Keywords:** capsule phase microextraction, ionic liquid, doxycycline, HPLC, urine

## Abstract

In this report, a polytetrahydrofuran-coated polyester fabric phase sorptive extraction (FPSE) for the determination of doxycycline in human urine was described. The sol-gel polytetrahydrofuran sorbent proved to be superior against other sol-gel coated cellulose and polyester membranes tested. The effect of the extraction parameters including membrane surface area, sample pH and volume, salt concentration, extraction time, stirring rate, etc., on the extraction efficiency of the analyte was studied using the “one-factor-at-a-time” (OFAT) and Box–Behnken design approaches. The analytical method proposed was validated in compliance with FDA guidelines for bioanalytical procedures. The method was linear in the determination range of 100–5000 ng/mL with the determination coefficient of 0.9953. The limit of detection (LOD) and the lower limit of quantification for doxycycline was 17 and 100 ng/mL, respectively. The relative recoveries for intra-day and inter-day studies ranged from 98.5–112.2% and 89.6–96.8%, respectively. The relative standard deviation was lower than 14.7% in all cases, exhibiting good precision. The sol-gel polytetrahydrofuran-modified FPSE membranes were reusable for at least 30 times. The greenness of the developed method was evaluated using Sample Preparation Metric of Sustainability (SPMS) and Blue Applicability Grade Index (BAGI) metric tools. Finally, the analytical scheme was successfully employed for the quantitation of urinary doxycycline collected at various time points following the administration of doxycycline-containing tablets.

## 1. Introduction

Antibiotics are substances either produced by microorganisms or synthesized to have potent antibacterial properties. They are primarily used as antibacterial agents in the treatment of infectious diseases in humans and animals [1]. Doxycycline (DOX) is considered one of the broadest-spectrum antibiotics. It has better tissue penetration than homologous antibiotic compounds (i.e., oxytetracycline, chlortetracycline), especially toward respiratory tissues [2,3]. The quantitation of certain drugs in biological fluids is essential as high concentration levels may lead to bacterial resistance and serious health problems such teeth coloring, allergic reactions, etc. [4].

When it comes to analysis of biofluids, a sample preparation step is typically required to remove the matrix interferences such as proteins, peptides, enzymes, salts, etc. [5]. The most popular sample preparation techniques employed in bioanalysis involve protein precipitation, liquid-liquid extraction (LLE), and solid-phase extraction (SPE). However, these approaches have several drawbacks involving high chemical consumption, obstacles in automation, and tedious sample preparation steps. To fill this gap, various liquid-phase and sorbent-based microextraction techniques have been developed in the last decades including liquid phase microextraction (LPME), fabric phase sorptive extraction (FPSE), solid-phase microextraction (SPME), stir bar sorptive extraction (SBSE), capsule phase microextraction (CPME), and pipette-tip solid-phase extraction (PT-SPE) [6]. The above techniques effectively comply with the general principles of Green analytical chemistry (GAC) such as the reduced organic solvent and sample consumption [7].

Several analytical methods have been reported for the determination of DOX in biological fluids [8,9,10,11,12]. These protocols comprise vortex-assisted hydrophobic ferrofluidic deep eutectic solvent dispersive liquid–liquid microextraction [8], protein precipitation with HClO_4_ [9], or acetonitrile [11], liquid–liquid extraction (LLE) with ethyl acetate [10], and dilution with water [12]. Ethyl acetate and HClO_4_ are non-green solvents while the ferrofluidic deep eutectic solvent requires synthesis and characterization. The analytical characteristics of the above approaches are summarized in Table S1.

The FPSE was initially proposed by Kabir and Furton a decade ago [13], as a promising and versatile sample preparation technique. The extraction efficiency and selectivity of this technique originated due to the usage of the organic polymer/organic-inorganic hybrid polymer, the organically modified inorganic precursor(s), and the fabric substrate. Different FPSE sorbents have been synthesized including neutral, cation or anion exchanger, mixed mode, graphene, and mixed-mode zwitterionic ones, and are utilized for the isolation of various analytes from complicated matrices [14,15]. Some advantages of FPSE include ease of handling, rapid-extraction kinetics, high-extraction efficiency, and an environmentally friendly profile.

To our knowledge, no validated FPSE-based method has been published in the literature for the determination of the urinary DOX. In this research, a simple FPSE procedure was developed for the quantitation of the drug in human urine. The experimental parameters of the FPSE protocol were studied and optimized using the “one-factor-at-a-time” (OFAT) and the Box–Behnken design (BBD) approaches, respectively. The analytical method was validated based on the international Food and Drug Administration (FDA) guidelines. The greenness of the FPSE-HPLC method was evaluated using SPMS and BAGI metric tools that considers the main principles of GAC [7,16]. The analytical protocol was employed for the monitoring of the urinary DOX concentration levels after the oral administration of the drug-containing pharmaceutical formulation.

## 2. Results and Discussion

### 2.1. Optimization of FPSE Conditions

#### 2.1.1. Effect of FPSE Membrane Type and Its Surface Area

The FPSE membrane type is critical and affects the extraction performance of the analyte. The mechanism relied on the sorbent’s sponge-like structure, which allows its functional groups to interact with the analyte through various interactions, including dipole-dipole interactions, London dispersion forces, π–π interactions, and hydrogen bonding.

**Figure 1:**
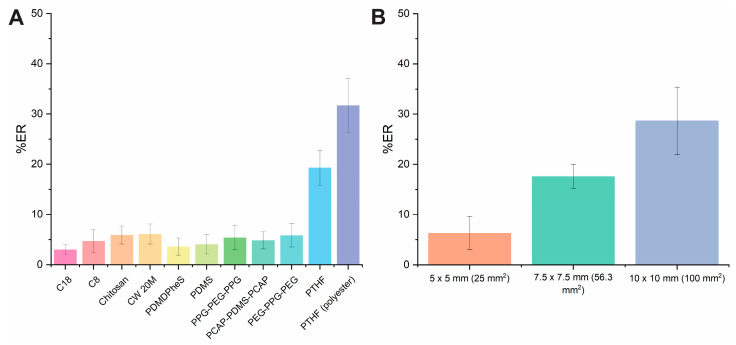
Effect of (**A**) FPSE membrane type and (**B**) FPSE surface area on the %ER of DOX.

Different sol-gel cellulose-based membranes with various polarity and functional groups were examined such as C_18_, C_8_, chitosan, CW 20M, PDMDPheS, PDMS, PPG-PEG-PPG, PCAP-PDMS-PCAP, PEG-PPG-PEG, and PTHF. Polyester-based PTHF was also studied. All FPSE membranes were cut to the appropriate size (10 × 10 mm). Each extraction experiment was conducted in triplicate (*n* = 3). A standard solution of DOX in artificial urine was used. The initial extraction parameters were as follows: sample pH, 6.8; no salt addition; extraction time, 45 min; stirring rate, 300 rpm; desorption time, 10 min; sample volume, 1000 μL; elution solvent, methanol (MeOH); elution volume, 1000 μL. The experiments indicated (Figure 1A) that the medium polarity sol-gel PHTF-based sorbent provided higher extraction recovery against other FPSE materials. This fact is mainly attributed to the presence of dipole–dipole interactions and hydrogen bonding of the PTHF skeleton and the hydroxyl groups of DOX (Figure S1, Supplementary Material). Moreover, the substrate plays a significant role in the retention of the DOX scaling from hydrophilic (100% cellulose) to hydrophobic (polyester) changing the overall performance. Finally, the sol-gel PTHF modified polyester membrane resulted in the highest %ER value (i.e., 30%) and was used for subsequent experiments.

Three different dimensions (i.e., 5 × 5, 7.5 × 7.5, and 10 × 10 mm) with active surface areas of 25, 56.25, and 100 mm^2^ were tested. As expected (Figure 1B), higher surface areas resulted in enhanced %ER of the analyte. Surface areas greater than 100 mm^2^ were not tested due to the geometrical features of the extraction vial. Finally, the dimension of 10 × 10 mm sol-gel coated PTHF polyester FPSE membranes was selected.

#### 2.1.2. Effect of Sample pH and Salt Concentration

The pH of the sample plays an important role in the extraction efficiency of the ionizable analyte as it influences the polarity and, therefore, its retention on the FPSE sorbent. The sample pH was investigated at the value of 2.5 and 6.8 (unadjusted). Alkaline conditions were not examined to avoid potential hydrolysis of the analyte [17]. Higher %ER values (ca 40%) were recorded at acidic conditions compared to neutral ones. It should be noted that the DOX molecule has three p*K*_a_ values of 3, 8, and 9.2 and, therefore, it will be predominately in its protonated form (more polar) at acidic conditions [18]. On this basis, the drug molecule interacts to a higher extent with the medium polarity PTHF-modified FPSE membrane. Thus, the sample pH value of 2.5 was chosen for further experiments.

**Figure 2:**
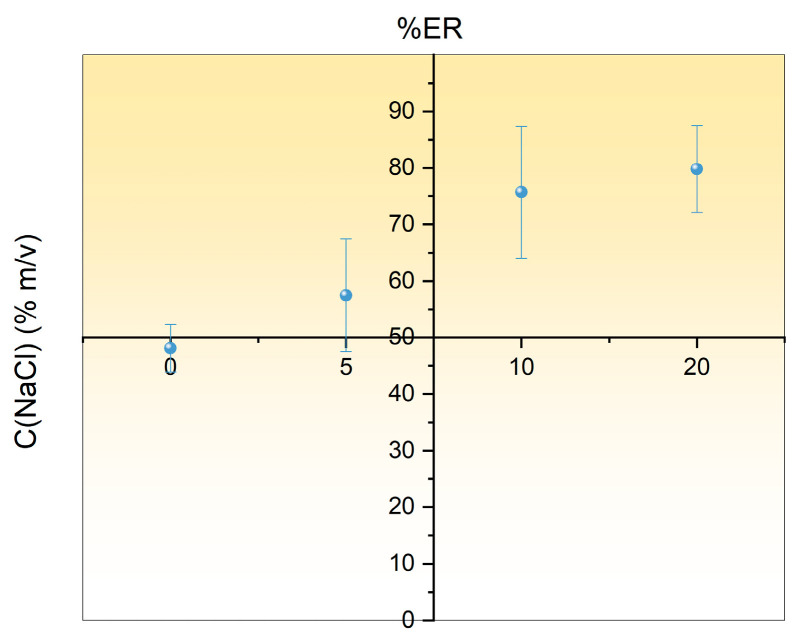
Effect of NaCl concentration on the %ER of DOX.

Salt addition can reduce the solubility of the analyte with intermediate polarity, resulting in its improved mass transfer to the FPSE membrane due to the salting-out effect. On the other hand, adverse effects may also occur since an increase in the solution viscosity can result in reduced mass transfer. The influence of various NaCl concentrations (i.e., 0–20% *w*/*v*) on the ER% of DOX was tested. As depicted in Figure 2, the ER% of the DOX was progressively increased by increasing the ionic strength of the sample up to 10% *w*/*v* and leveled-off thereafter. Analogous results have been also reported by E. A. Dil [8]. Based on these findings, the NaCl concentration of 10% *m*/*v* was chosen for the FPSE protocol.

#### 2.1.3. Effect of the Extraction Time, Stirring Rate, and Sample Using Box-Behnken Design

The extraction time, the sample volume, and the stirring rate were thoroughly studied and optimized using response surface methodology. For this purpose, a Box-Behnken design (BBD) was built using the Design Expert^®^ 13 software (Stat-Ease^®^ Inc., Minneapolis, MN, USA) and was used for 17 experiments. These runs included 12 axial and factorial and 5 center point experiments. The experimental domain and the obtained %ER values were tabulated in Table 1. The potential systematic errors were eliminated by randomizing the experiments. For the construction of the fitted second-order polynomial quadratic model, multivariate regression analysis was employed. The non-significant parameters were excluded using the backward elimination (*p* > 0.05). The obtained models were statistically validated.

**Figure 3:**
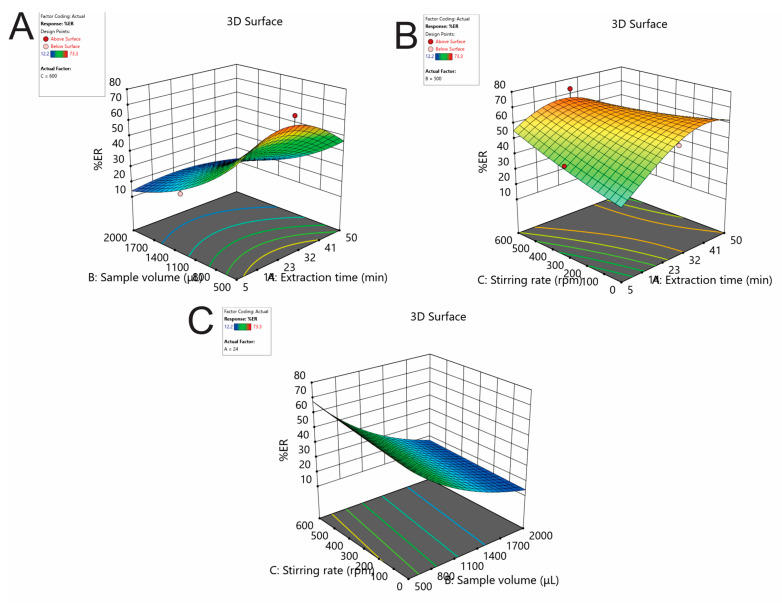
Response surface plots showing the effects of (**A**) sample volume and extraction time, (**B**) stirring rate and extraction time, and (**C**) stirring rate and sample volume on the %ER of DOX.

The %ER outputs were logarithmically transformed (log10) to achieve a normal distribution of the residuals. The ANOVA results are tabulated in Table S2. As it was obtained, the lack-of-fit (LOF) of the model was non-significant. The *R*^2^ value was 0.9254, exhibiting satisfactory reliability and predictability. The diagnostic plots are depicted in Figure S2 (Supplementary Material). A strong correlation between the predicted and actual responses was observed, with all data points consistently aligned around the trend line. The response surface and contour plots of the analyte are illustrated in Figure 3. Curvature was observed in all plots.

The optimal FPSE experimental conditions were found utilizing Derringer’s desirability function (D). The counter plots of the desirability function are shown in Figure S3 (Supplementary Material). After rounding of the optimal experimental values, the optimum conditions were an extraction time of 24 min, sample volume of 500 μL, and stirring rate of 600 rpm. To confirm the optimum extraction conditions, six repetitive extractions (*n* = 6) were carried out. The variations between the predicted and the experimental values were found to be satisfactory (<7%).

#### 2.1.4. Effect of the Elution Solvent, Volume and Time

**Figure 4:**
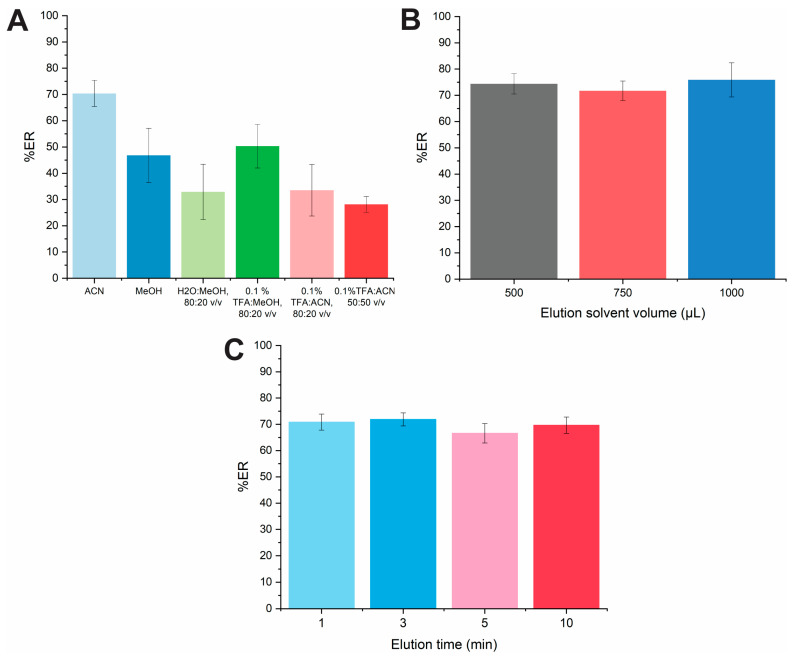
Effect of the (**A**) elution solvent type, (**B**) elution solvent volume on the %ER and (**C**) elution time of DOX.

The study of the elution conditions is essential to ensure the effective elution of DOX. Six different solvents were tested including ACN, MeOH, H_2_O:MeOH 80:20 *v*/*v*, 0.1% TFA:MeOH 80:20 *v*/*v*, 0.1% TFA:ACN 50:50 *v*/*v*, and 0.1% TFA:ACN 80:20 *v*/*v*. As portrayed in Figure 4A, the mixture of 0.1% TFA or water with MeOH or ACN resulted in almost similar %ER values (range 30–50%) indicating that the drug cannot be quantitatively eluted from the FPSE membrane due to the potential existence of hydrogen bonding and van der Waals interactions between the PTHF-modified FPSE material and the drug molecule. However, the highest %ER value was recorded using pure ACN as it has higher eluotropic strength than other tested solvents. For this reason, this solvent was finally chosen as the elution solvent and adopted for further experiments. The influence of the desorption solvent volume on the %ER of the drug was examined in the range of 500–1000 μL (Figure 4B). It was observed that the volume of ACN used for the elution of DOX had a non-significant impact on its %ER. Thus, an aliquot of 500 μL was selected for further studies to avoid sample dilution.

The elution time was studied in the time span of 1–10 min. The %ER was not statistically different at the examined intervals (Figure 4C). Adequate desorption of DOX was achieved even at 1 min and this time period was chosen for further experiments ensuring improved sample throughput.

### 2.2. Method Validation

**Figure 5:**
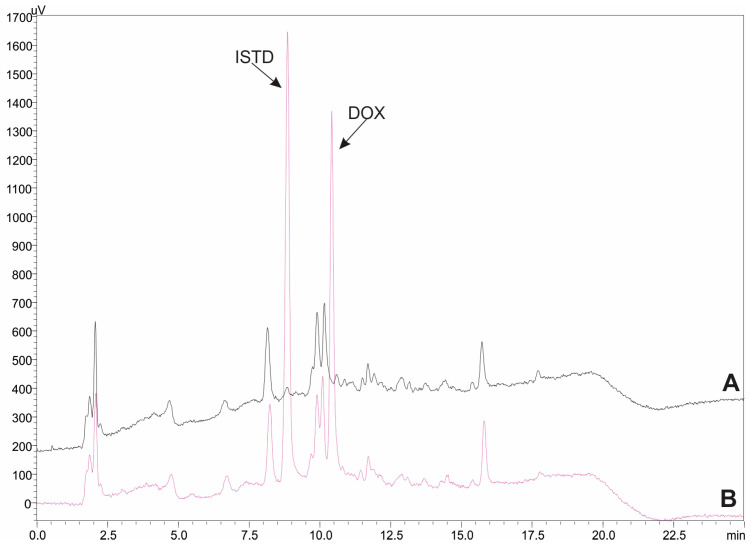
Representative HPLC-UV chromatograms for the analysis of (A) blank urine sample, (B) blank urine sample spiked with DOX and ISTD at concentration level of 2000 ng/mL and 2500 ng/mL, respectively.

The selectivity of the bioanalytical method was tested by analyzing different human blank urine samples and spiked at concentration level of 2000 ng/mL. The respective chromatograms are illustrated in Figure 5. No peaks that could interfere with the determination of the DOX and the internal standard (ISTD) were detected at the retention time of the compounds. To evaluate the potential carry-over effect, an injection of blank sample was performed after the analysis of a spiked sample at the highest calibrated level of 5000 ng/mL. The recorded signals were below 5% of those of the LLOQ level. As a result, the column, the FPSE membrane, and the autosampler were thoroughly cleaned between analyses.

Method linearity was assessed using standards in neat solvent and standard addition in the concentration range of 100 to 5000 ng/mL. The ratio of the peak areas of DOX and ISTD was used as the response to evaluate the linearity of the method. The determination coefficients of the unweighted linear regressions in neat solvent and standard addition were higher than 0.9953, demonstrating good linearity (Figure S4, Supplementary Material).

In the analysis of biological samples using equilibrium-based micro-extraction techniques, the extraction performance of the analyte might be diminished compared to that in aqueous solutions due to a shift in the extraction kinetics [19]. To investigate this parameter, the slopes of the regression equations were compared. The slope ratio was found to be 1.08 for the DOX, exhibiting negligible matrix effect. Therefore, the neat solvent calibration can be utilized for the quantification of the analyte in the samples. The lower limit of quantitation (LLOQ) was set at 100 ng/mL and the limit of detection (LOD) (based on the *S*/*N* = 3 criterion) was estimated to be 17 ng/mL.

Τhe relative standard deviation (RSD%) values ranged from 3.3–10.3% and 3.7–14.7% for the intra-day and inter-day precision, respectively. The relative recovery (%RR) values for intra-day and inter-day accuracy were between 89.6 and 112.2%. The values mentioned are tabulated in Table 2. 

The robustness of FPSE protocol was examined using Plackett-Burman design (Table S3). This design was created using the TIBCO Statistica^®^ software v. 13.3.0 (TIBCO software Inc. Palo Alto, CA, USA) examining only the main effects of these parameters. The interpretation of the effects on the responses was carried out graphically (Pareto charts). As shown in Figure S5, none of the examined parameters was statistically significant (*p* > 0.05, significant level 5%) on the peak area of the DOX and the ISTD revealing that the proposed sample preparation protocol is robust.

**Figure 6:**
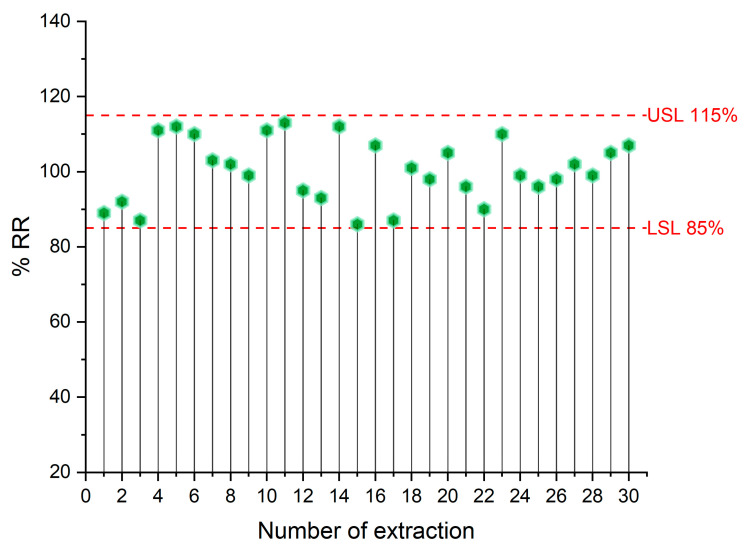
Reusability of the PTHF-based FPSE membrane.

The reusability of the sol-gel PTHF-modified polyester membranes was assessed by repeatedly extracting DOX from a spiked urine sample. A 15% reduction in extraction efficiency (measured as % relative recovery) was set as the criterion. The experiments demonstrated that the FPSE membrane can be used at least 30 times. (Figure 6).

### 2.3. Analysis of Real Samples

The proposed FPSE method was evaluated by analyzing authentic human urine samples obtained from a volunteer who underwent DOX treatment. The samples were collected at pre-determined time points (2, 4, 6, 8, 10, and 12 h) after oral administration of DOX-containing formulation (Vibramycin^®^ 100 mg/tab, Pfizer, New York, USA). A set of representative HPLC chromatograms is depicted in Figure S6 (Supplementary Material). The urinary DOX profile illustrated is in Figure S7. Analogous findings have been published by Haaland et. al. [20]. The developed protocol proved effective for determining the specific drug and could be a valuable tool for supporting DOX pharmacokinetic studies.

### 2.4. Evaluation of Method’s Greenness Using SPMS and BAGI Metric Tools

**Figure 7:**
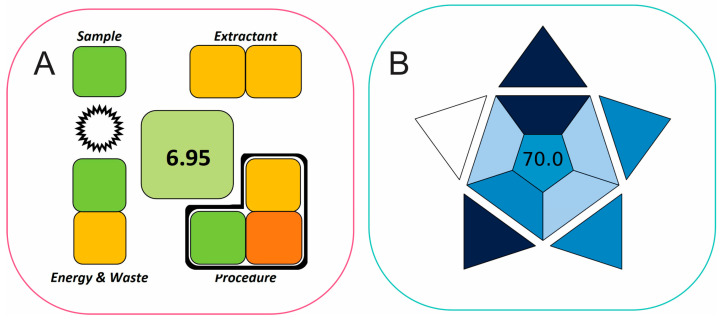
(**A**) SPMS clock diagram and (**B**) BAGI pictogram.

The method’s greenness and applicability were assessed using SPMS [21] and BAGI [22] metric tools. The SPMS metric evaluates the greenness of sample preparation techniques by assessing the entire analytical procedure, including sampling, sample preparation, and final detection and quantitation. This metric can explicitly and exclusively evaluate the sample preparation, and it reports the result with a clock-like diagram, displaying the greenness evaluation of the main experimental parameters and a total score (Figure 7A). The proposed sample preparation method demonstrates a green character (score of 6.95), indicating reduced chemical consumption and waste generation, and reusable extractant during the FPSE microextraction step.

The BAGI, a recently introduced tool, offers a quantitative method for evaluating the “blueness” or suitability of analytical methods. “Blueness” refers to how well an analytical technique meets practical criteria and is fit-for-purpose in real-world applications [22]. The generated BAGI pictogram for the proposed CPME method is shown depicted in Figure 7B. A score of 70.0 was achieved, demonstrating adequate method applicability. This result can be attributed to high sample throughput, simple instrumentation and required reagents, and good sensitivity that eliminates the need for further preconcentration.

## 3. Materials and Methods

### 3.1. Reagents, Solutions, and Materials

All reagents were of analytical grade or higher unless otherwise stated. The DOX hydrochloride (>99%), acetonitrile (ACN) and MeOH, and trifluoroacetic acid (TFA) were purchased from Sigma-Aldrich (St. Louis, MO, USA). Moxifloxacin—used as ISTD—was obtained from its commercially available pharmaceutical formulation (Mikrobiel^®^ 1.6 mg/mL, Cooper Pharmaceuticals, Athens, Greece). High-purified water (Milli-Q) was provided by a B30 system (Adrona SIA, Riga, Latvia).

Stock standard solutions of DOX and ISTD (1000 μg/mL each) were individually prepared in methanol and water, respectively, and kept at 4 °C. Diluted standard solutions and mixtures were prepared in water.

Artificial urine was prepared by dissolving lactic acid (0.05 g), citric acid (0.2 g), sodium bicarbonate (1.05 g), urea (5 g), CaCl_2_·2H_2_O (0.185 g), NaCl (2.6 g), MgSO_4_·7H_2_O (0.245 g), Na_2_SO_4_·10H_2_O (0.475 g), KH_2_PO_4_ (0.475 g), K_2_HPO_4_ (0.6 g), and NH_4_Cl (0.65 g) in 500 mL H_2_O. The pH of the resulting solution was adjusted to 6.5 by addition of 1.0 mol/L H_3_PO_4_ [23].

The FPSE media were manufactured in the Department of Chemistry and Biochemistry at Florida International University (Miami, FL, USA). Unbleached muslin cotton (100% cellulose) and polyester were used as fabric substrates and were supplied by Jo-Ann Fabric (Miami, FL, USA). Sol-gel precursors, triblock copolymers, graphene, CF_3_COOH (TFA), CH_3_COCH_3_, CH_2_Cl_2_, NaOH, HCl, methyl trimethoxysilane (MTMS), tetramethoxysilane (TMOS), and isopropanol (IPA) were provided by Sigma-Aldrich (St. Louis, MO, USA) and Glest Inc. (Morrisville, PA, USA).

### 3.2. Fabrication of FPSE Membranes

Due to the structural characteristics of the analyte (see Figure S1), it is challenging to predict the most suitable FPSE sorbent material that would effectively isolate the analyte in the presence of interfering compounds. Based on this fact, a set of FPSE membranes were prepared. The characteristics of FPSE membranes are tabulated in Table S4. More details about the preparation and characterization of FPSE membranes can be found elsewhere [24].

### 3.3. Instrumentation and HPLC Conditions

All separations were carried out using a Shimadzu HPLC-UV system (Kyoto, Japan) comprising two high pressure binary gradient pumps (LC-20AD), an SPD-20A UV detector, an SIL-10AD autosampler, and a CBM-20A controller. The HPLC operation and data processing were performed using LC Solutions software (version 1.25 SP4). The separation of DOX and the ISTD was performed on an ACE C_18_ analytical column (150 × 4.6 mm, 5 μm) from VMR International (Leicestershire, UK). The separation was accomplished using a gradient program using water (mobile phase A, MP_A_) and acetonitrile (mobile phase B, MP_B_) both acidified with 0.1% *v*/*v* TFA. The initial %B content was 20% which was linearly increased to 60% at 15 min and returned to its initial value (20%) at 17 min. The stationary phase was equilibrated at the initial composition until 25 min. The flow rate and the injection volume were 1 mL/min and 10 μL, respectively. All separations were performed at ambient temperature. Both analyte and ISTD were monitored at 345 nm.

An MR82 magnetic stirrer (Heidolph Instruments GmbH & Co. KG, Schwabach, Germany) and a K-550-GE vortex (Scientific Industries, Inc., New York, NY, USA were used throughout this study.

### 3.4. Sample Collection

Human urine samples were collected from healthy volunteers who were fully informed about the research purpose. All samples were initially centrifuged at 5000 rpm for 10 min, and the supernatant was stored at −18 °C. Prior to analysis, 250 μL of each sample was combined with 50 μL of the ISTD solution and 200 μL of the DOX standard solution (or water for the blank) in an extraction vial, followed by vortex mixing to ensure sample homogeneity.

### 3.5. FPSE Procedure

**Figure 8:**
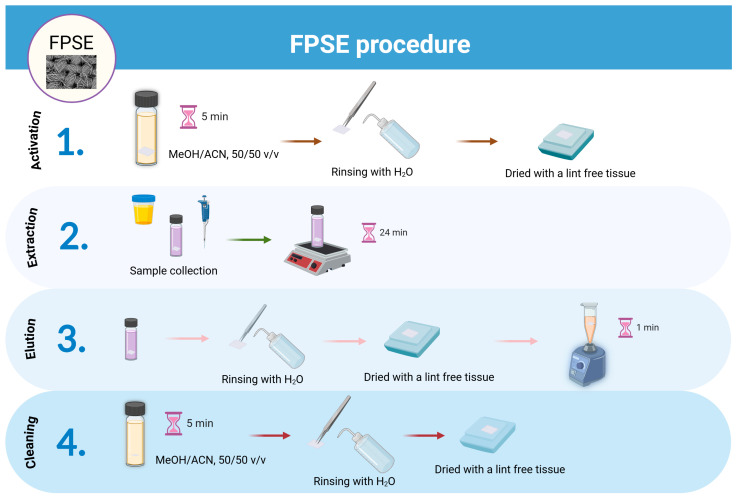
Main steps of the proposed FPSE protocol (Created with BioRender.com).

The FPSE steps are illustrated in Figure 8 and included the following subsequent steps:

(i) Activation: Initially, the FPSE membrane (10 mm × 10 mm) was activated with a 1 mL mixture of MeOH/ACN, 50:50 *v*/*v* for 5 min. Then, the device was rinsed with water to remove any solvent traces that might reduce the performance of the extraction process. Then, the device was dried with a lint-free tissue.
(ii) Extraction: 500 μL of sample was transferred to a cylindrical vial (65 × 15 mm) with the FPSE membrane. The extraction of the analyte was performed under constant stirring at 600 rpm for 24 min.
(iii) Elution: After the extraction step, the FPSE membrane was rinsed with water to remove any sample residues left on the surface was dried with a lint-free tissue. Then, it was placed into an Eppendorf tube containing 500 μL ACN followed by vortex for 1 min.
(iv) Cleaning: The FPSE membrane was then immersed in the mixture of MeOH/ACN 50:50 *v*/*v* for cleanup and stored until its next use.

### 3.6. Method Validation

The proposed FPSE-HPLC method was validated according to the FDA guidelines [25]. The selectivity was examined by analyzing different blank and spiked authentic human urine samples. The carry-over effects were examined by analyzing blank samples after spiked samples at the highest concentration level of 5000 ng/mL. Τhe signals from the blank samples are considered significant if being more than 5% of those at LLOQ level.

The method linearity was evaluated by analyzing standards in artificial urine and standard addition in authentic urine. The peak area ratio (DOX/ISTD) versus the concentration of DOX was used. A pooled sample was spiked at seven concentration levels covering the range of 100 to 5000 ng/mL. Three independent extractions (*n* = 3) were made at each level. The matrix effect was investigated by comparing the parallelism of the external and the standard addition calibration curves. A slope ratio between 0.8 and 1.2 indicates negligible matrix effect. The LOD was estimated based on the *S*/*N* = 3 criterion. The LLOQ was set to 100 ng/mL.

The within-day method precision and accuracy were studied by three analyses at four different concentration levels (i.e., LLOQ, LQC, MQC, and HQC). The inter-day precision and accuracy were examined on three different consecutive days. The acceptance criteria for accuracy should be between 85 and 115% for all levels and 80 and 120% for LLOQ. The precision (expressed as %RSD) must be less than 15% or 20%, depending on LLOQ level.

## 4. Conclusions

Herein, an FPSE-HPLC-UV method was proposed for the determination of urinary DOX using sol-gel PTHF-modified polyester membranes. Among the advantages of the proposed analytical protocol are its handling simplicity, the relatively fast extraction kinetics, the high sample throughput, and the low cost of the microextraction media. Furthermore, the proposed analytical scheme exhibited multiple green aspects, as revealed by the SPMS and BAGI indexes. The developed method was successfully used in authentic urine analysis, showcasing its effectiveness for clinical applications in monitoring DOX urinary levels.

## Figures and Tables

**Table 1:** Box-Behnken design and the obtained %ER value of DOX for each run.

Run No	Extraction Time (min) (Factor 1)	Sample Volume (μL) (Factor 2)	Stirring Rate (rpm) (Factor 3)	%ER
1	27.5	500	0	57
2	50	1250	600	25.7
3	5	2000	300	12.2
4	27.5	500	600	73.3
5	5	1250	600	25.5
6	27.5	1250	300	27.5
7	50	500	300	46.8
8	27.5	1250	300	41.5
9	27.5	2000	0	15.6
10	5	1250	0	14
11	27.5	1250	300	22.5
12	50	1250	0	31.8
13	5	500	300	44.4
14	27.5	2000	600	13.8
15	50	2000	300	13.3
16	27.5	1250	300	35.8
17	27.5	1250	300	33.9

**Table 2:** Intra-day and inter-day precision and accuracy data of the FPSE-HPLC method for the determination of DOX in human urine.

	Intra-Day (*n* = 3)		Inter-Day (*n* = 3)	
Added Concentration (ng/mL)	Precision (%RSD)	Accuracy RR ^1^ (%)	Precision (%RSD)	Accuracy RR (%)
100 (LLOQ)	3.3	112.2	14.7	96.8
250 (LQC)	10.3	98.5	9.9	89.6
750 (MQC)	7.7	100.5	3.7	91.9
5000 (HQC)	8.4	99.1	4.0	95.2

^1^ RR: relative recovery.

## Data Availability

Data available on request due to restrictions.

## Supplementary Materials

The following supporting information can be downloaded at: https://www.mdpi.com/article/10.3390/molecules29174076/s1, Figure S1: Chemical structures of doxycycline (DOX) and moxifloxacin (used as internal standard).; Figure S2: Normal probability and the residuals vs predicted plots for the %ER of DOX.; Figure S3: Contour plots of desirability functions.; Figure S4: Calibration curves of DOX in artificial and pooled authentic urine sample using the proposed FPSE-HPLC method.; Figure S5: Pareto charts of the Plackett-Burman design for the robustness test of FPSE procedure for (A) DOX and (B) ISTD; Figure S6: Representative HPLC chromatograms for the analysis of authentic human urine sample collected after (A) 2 h, (B) 4 h, (C) 6 h, (D) 8 h, (E) 10 h, and (F) 12 h after administration of DOX-containing pharmaceutical formulation (Vibramycin^®^, 100 mg/tab).; Figure S7: Urinary DOX profile after oral administration of DOX-containing formulation (Vibramycin^®^ 100 mg/tab, Pfizer). Table S1: Comparison of the proposed FPSE-HPLC method with other reported approaches for the determination of the analyte in biological fluids.; Table S2: ANOVA table for the extraction recovery (%ER) of DOX.; Table S3: Plackett-Burman design for the robustness test of FPSE procedure.; Table S4: Characteristics of FPSE membranes.
