# Supplementary material for: Exploiting the Applicability of Polytetrahydrofuran-Modified Polyester for the Fabric Phase Sorptive Extraction of Doxycycline from Human Urine
Source: Molecules. 2024 Aug 28;29(17):4076. doi: 10.3390/molecules29174076 (PMC11397658; doi:10.3390/molecules29174076)
Supplement: Supplementary file 1 [file molecules-29-04076-s001.zip › molecules-3150295-supplementary.pdf]

SUPPLEMENTARY MATERIAL

Exploiting the applicability of polytetrahydrofuran-coated polyester for the fabric phase sorptive extraction of doxycycline from human urine

Panagiotis Chatzintounas[1], Marianna Ntorkou[1], Abuzar Kabir[2], and Constantinos K. Zacharis[1],*

[1]*Laboratory of Pharmaceutical Analysis, School of Pharmacy, Aristotle University of Thessaloniki, 54124 Thessaloniki, Greece*

[2]*Department of Chemistry and Biochemistry, Florida International University, Miami, FL, USA*

---

*Corresponding author
Constantinos K. Zacharis
Associate Professor
Laboratory of Pharmaceutical Analysis, Department of Pharmacy,
Aristotle University of Thessaloniki (AUTh),
GR-54124, Greece
Tel: +30 2310997663
E-mail: czacharis@pharm.auth.gr

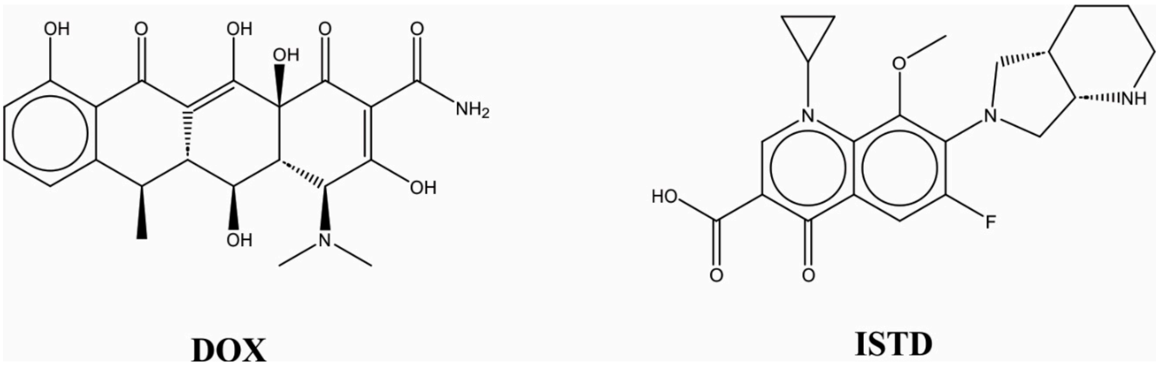

Figure S1. Chemical structures of doxycycline (DOX) and moxifloxacin (used as internal standard).

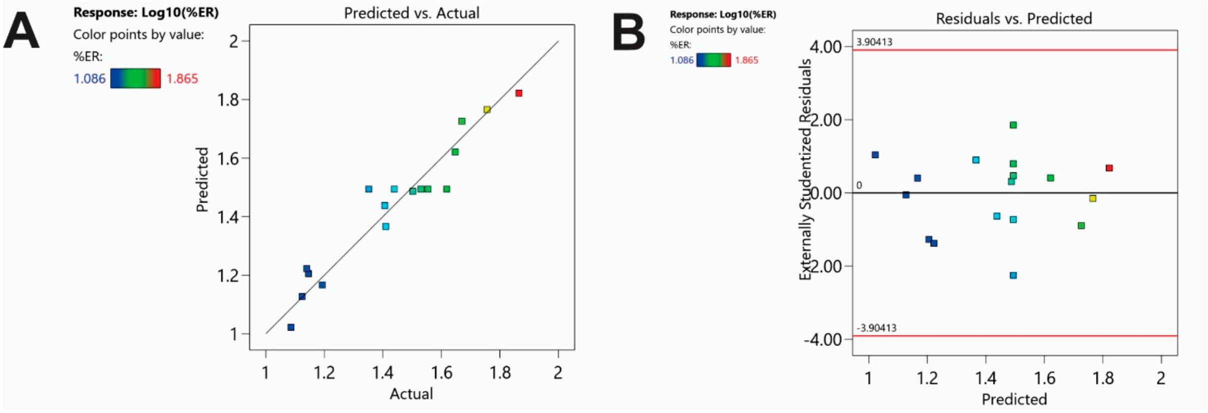

Figure S2. Normal probability and the residuals *vs* predicted plots for the %ER of DOX.

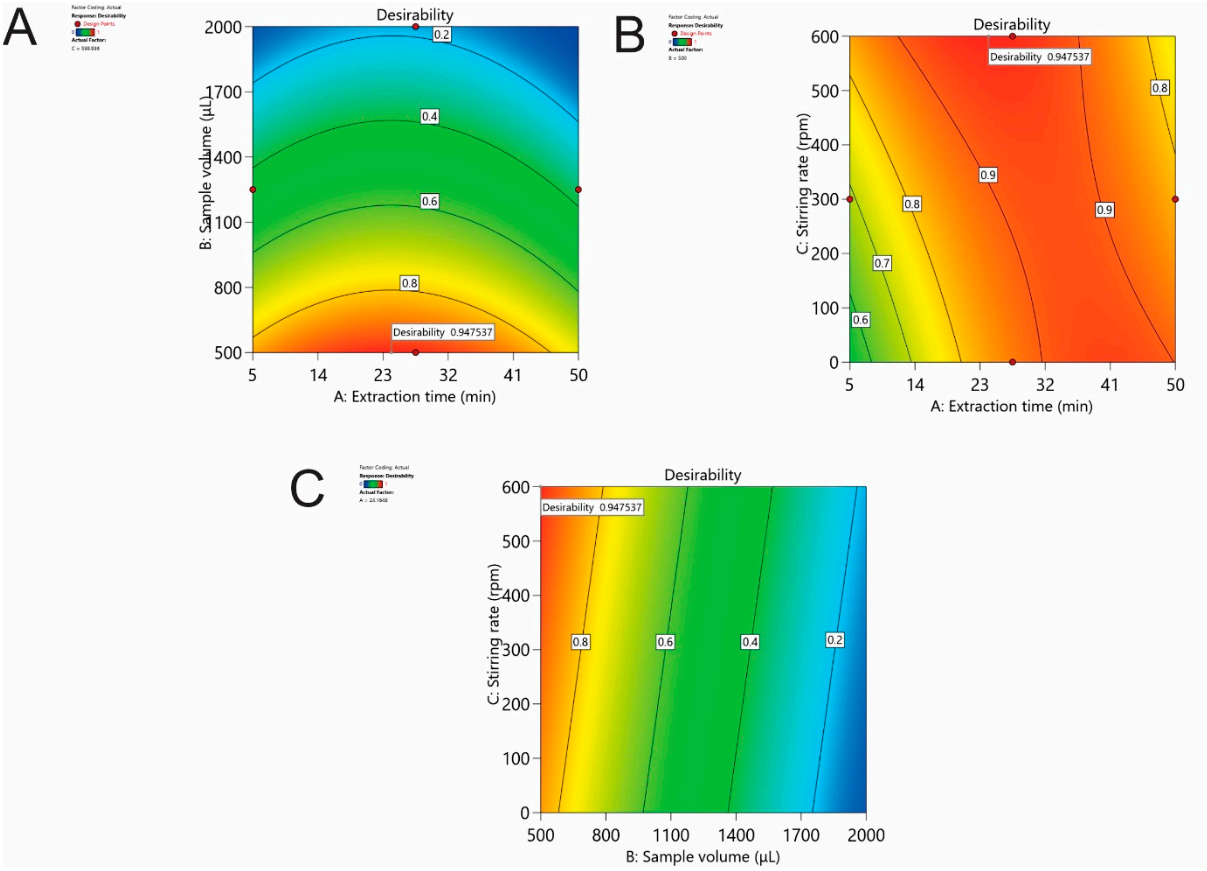

Figure S3. Contour plots of desirability functions.

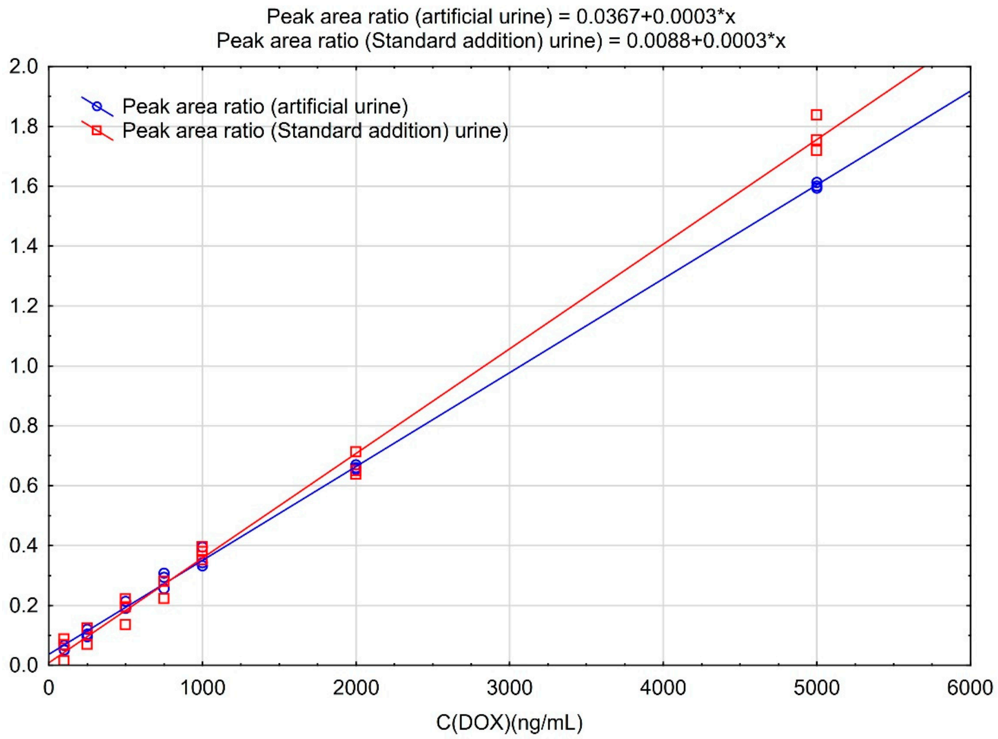

Figure S4. Calibration curves of DOX in artificial and pooled authentic urine sample using the proposed FPSE-HPLC method.

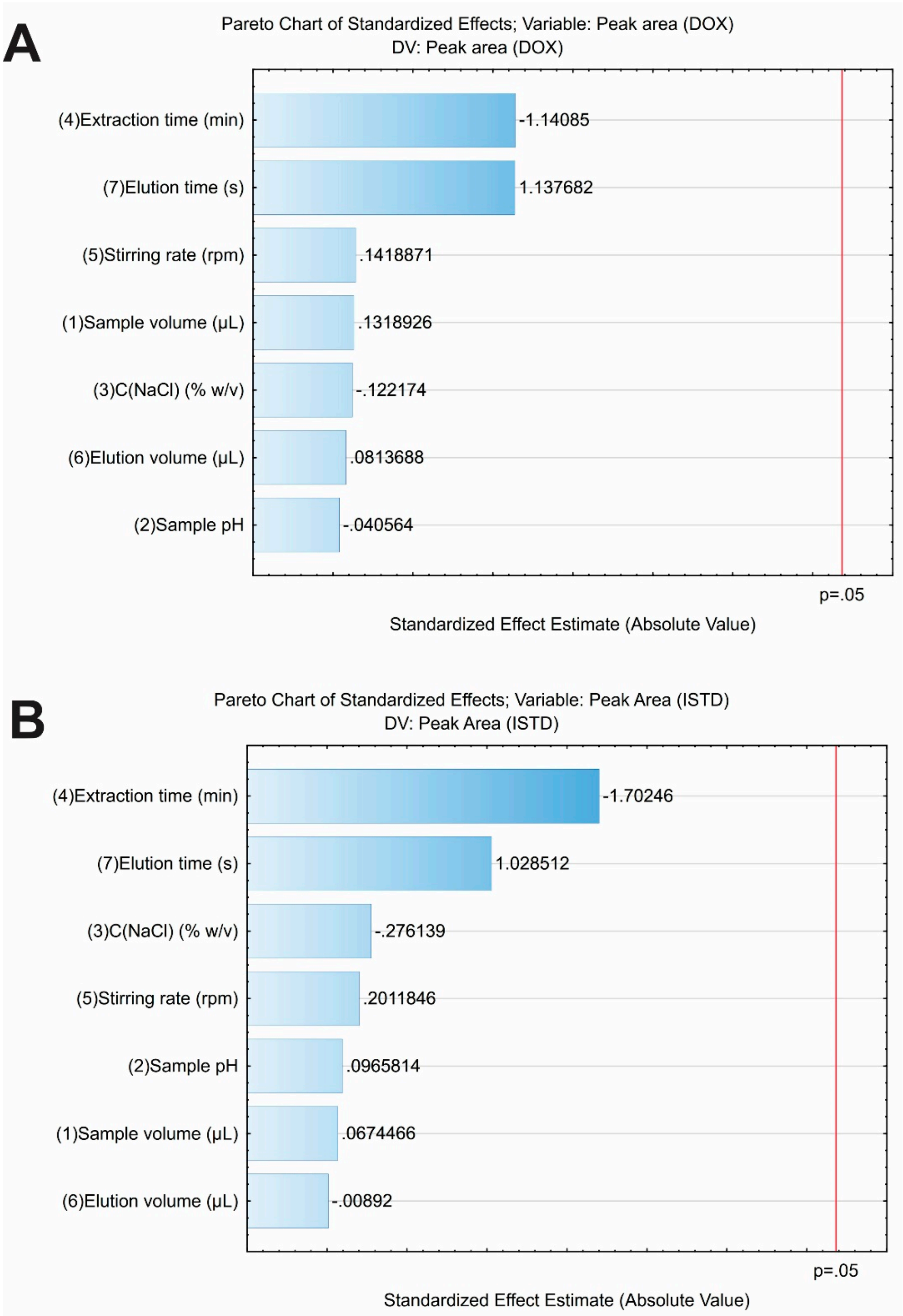

Figure S5. Pareto charts of the Plackett-Burman design for the robustness test of FPSE procedure for A) DOX and B) ISTD.

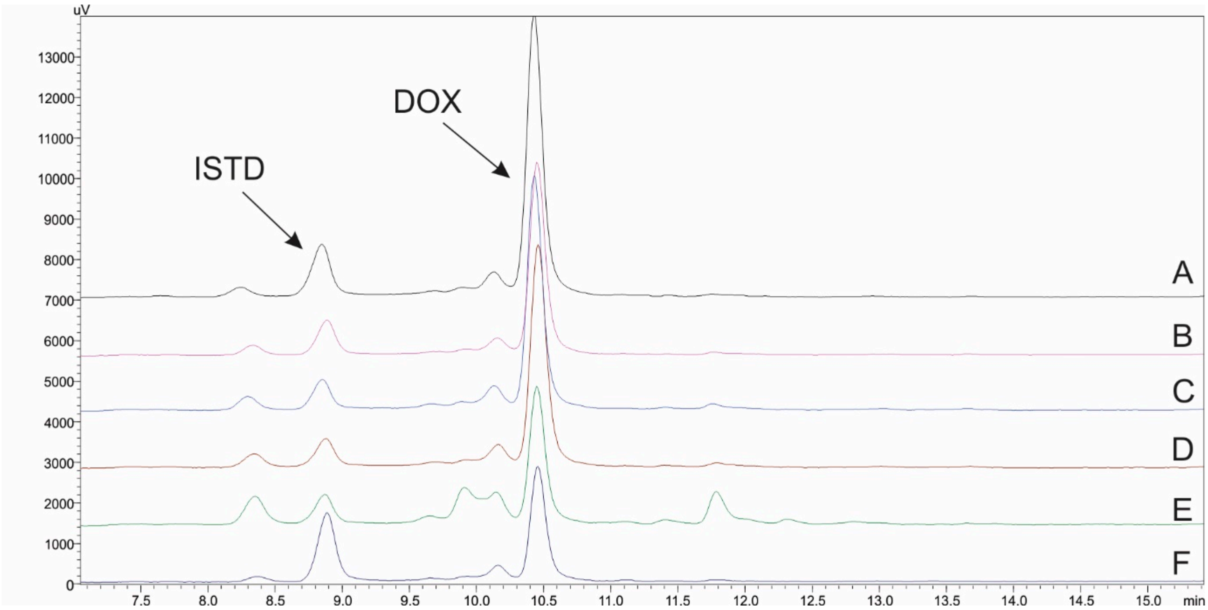

Figure S6. Representative HPLC chromatograms for the analysis of authentic human urine sample collected after A) 2h, B) 4h, C) 6h, D) 8h, E) 10h, and F) 12h after administration of DOX-containing pharmaceutical formulation (Vibramycin®, 100 mg/tab).

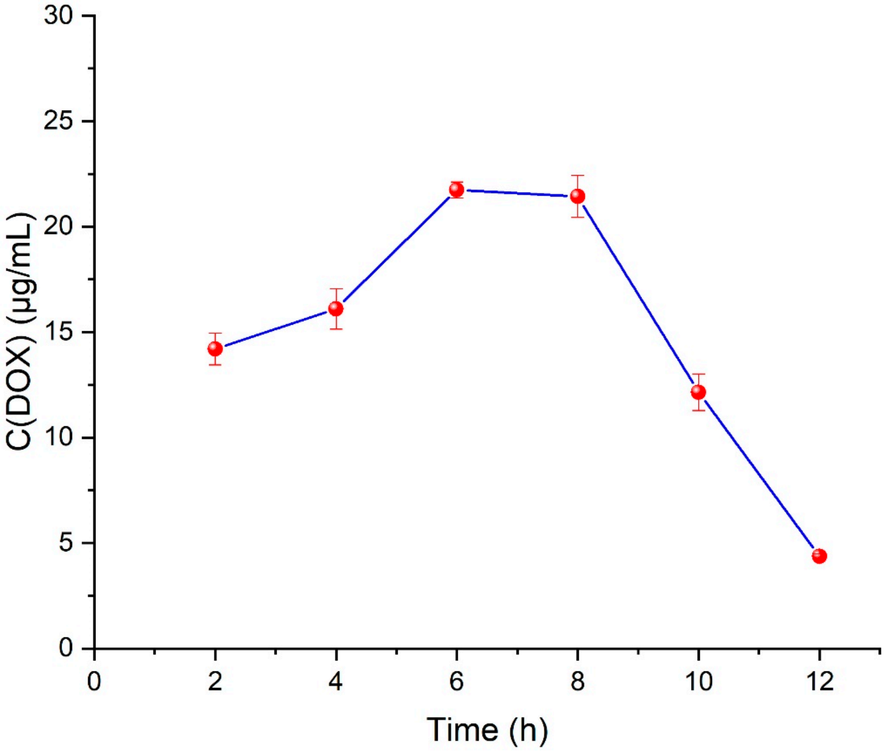

Figure S7. Urinary DOX profile after oral administration of DOX-containing formulation (Vibramycin® 100 mg/tab, Pfizer).

Table S1. Comparison of the proposed FPSE-HPLC method with other reported approaches for the determination of the analyte in biological fluids.

Sample Preparation	Extraction sorbent/reagent	Separation Technique	LOD (ng mL^{-1})	LOQ (ng mL^{-1})	Relative Recovery (%)	Ref.
Vortex assisted hydrophobic ferrofluidic deep eutectic solvent dispersive liquid liquid microextraction	Magnetic nanoparticles	HPLC-UV	3.6	8.5	86.7-97.5	[8]
Protein Precipitation	$HClO_4$	HPLC-UV	87	264	84.9 – 96.5	[9]
Liquid-liquid extraction	Ethyl acetate	LC-MS/MS	-	10	89.1 – 103.4	[10]
Protein precipitation	CH_3CN	HPLC-FLD	1.63 - 6.36	4.93 – 19.28	95.22 – 98.98	[11]
Dilute and shoot	water	CE-UV	100	500	95.1 – 96.7	[12]
**FPSE**	**Polytetrahydrofuran-modified FPSE membrane**	**HPLC-UV**	**17**	**100**	**89.6 – 112.2**	**This study**

Table S2. ANOVA table for the extraction recovery (%ER) of DOX.

Source	Sum of Squares	df[1]	Mean Square	*F*-value	*p*-value	
**Model**	0.8378	5	0.1676	27.31	< 0.0001	significant
A-Extraction time	0.0221	1	0.0221	3.59	0.0845	
B-Sample volume	0.7172	1	0.7172	116.89	< 0.0001	
C-Stirring rate	0.0063	1	0.0063	1.02	0.3339	
AC	0.0311	1	0.0311	5.07	0.0457	
A^2	0.0611	1	0.0611	9.95	0.0092	
**Residual**	0.0675	11	0.0061			
Lack of Fit	0.0242	7	0.0035	0.3198	0.9102	not significant
Pure Error	0.0433	4	0.0108			
**Cor Total**	0.9053	16				

[1]degree of freedom

Table S3. Plackett-Burman design for the robustness test of FPSE procedure.

Run No	Sample volume (μL)	Sample pH	C NaCl (% w/v)	Extraction time (min)	Stirring rate (rpm)	Elution volume (μL)	Elution time (sec)	Peak Area (DOX)	Peak Area (ISTD)
1	500	2.55	10	24	600	500	60	33753	18247
2	510	2.6	11	26	610	510	70	20560	9816
3	500	2.55	10	24	600	500	60	33524	18408
4	510	2.5	9	22	590	510	70	28987	16364
5	490	2.5	9	26	610	510	50	12531	6246
6	490	2.5	11	26	590	490	70	18278	8428
7	490	2.6	11	22	590	510	50	18597	11395
8	510	2.6	9	26	590	490	50	11574	6136
9	500	2.55	10	24	600	500	60	26341	14667
10	490	2.6	9	22	610	490	70	28175	17296
11	510	2.5	11	22	610	490	50	20287	12100

Table S4. Characteristics of FPSE membranes.

FPSE material	Fabric substrate	Polarity
Sol-gel PEG-PPG-PEG	Cellulose	Medium polar
Sol-gel PPG-PEG-PPG	Cellulose	Medium polar
Sol-gel PTHF	Cellulose	Medium polar
Sol-gel CW 20M	Cellulose	Polar
Sol-gel PDMS	Cellulose	Non-polar
Sol-gel C_{18}	Cellulose	Non-polar
Sol-gel Chitosan	Cellulose	Polar
Sol-gel C_8	Cellulose	Non-polar
Sol-gel PCAP-PDMS-PCAP	Cellulose	Medium polar
Sol-gel PDMDPheS	Cellulose	Non-polar
Sol-gel PTHF	Polyester	Medium polar
